# Correction: A nuclear role for the DEAD-box protein Dbp5 in tRNA export

**DOI:** 10.7554/eLife.63264

**Published:** 2020-09-24

**Authors:** Azra Lari, Arvind Arul Nambi Rajan, Rima Sandhu, Taylor Reiter, Rachel Montpetit, Barry P Young, Chris JR Loewen, Ben Montpetit

Lari A, Rajan AAN, Sandhu R, Reiter T, Montpetit R, Young BP, Loewen CJR, Montpetit B. 2019. A nuclear role for the DEAD-box protein Dbp5 in tRNA export. *eLife* **8**:e48410. doi: 10.7554/eLife.48410.

Published 27, August 2019

We have identified a mutant strain used in this study that is not the correct genotype. Based on our records, the *dbp5-L12A-R423A* strain was generated at the same time as a number of other mutant strains and DNA sequencing of two independent isolates showed the correct genotype. These strains were then stored as glycerol stocks and used in the study. In follow on work after publication, we introduced the *dbp5-L12A-R423A* mutation in to an alternate genetic background and resulting strains were found to have unexpected phenotypes. This ultimately led to resequencing *dbp5-L12A-R423A* from the original glycerol stocks and it was found that the genotype was incorrect. We do know how this mistake arose, but suspect that strains were inadvertently mixed up at a point following sequencing and prior to the strains being frozen as glycerol stocks. It is our opinion that the true phenotypes of the *dbp5-L12A-R423A* mutant do not alter major conclusions made in the paper. We sincerely regret this error and apologize to anyone impacted by our mistake. We have corrected the manuscript through the following changes:

1. The manuscript text has been corrected to reflect figure changes and to report the correct mutant phenotype of *dbp5-L12A-R423A*. Original and corrected text are shown below with new text indicated in bold.

Original:

- Importantly, a strain expressing GFP-Dbp5^L12A/R423A^ is viable and there is an increased nuclear pool of Dbp5 as compared to R423A (Figure 4—﻿figure supplement 1A). Further, the untagged and integrated version of *dbp5-L12A/R423A* does not exhibit mRNP export or temperature-dependent growth defects (Figure 4—﻿figure supplement 1B-C). Using the double mutant, northern blotting was performed with probes against the tRNA^Ile^_UAU_ at 37°C (Figure 4D). As compared to *dbp5-R423A* (2.2 ± 0.2), accumulation of the intron containing tRNA intermediate was reduced in the integrated *dbp5-L12A/R423A* (1.4 ± 0.3) strain to a level comparable to *dbp5-L12A* (1.4 ± 0.2), suggesting that increasing the nuclear pool of Dbp5 through inclusion of the L12A mutation largely rescued tRNA processing defects related to the R423A mutation. Combined, the SGA, northern, and in situ data employing the *dbp5-L12A* and *dbp5-R423A* mutations to alter Dbp5 nuclear levels are strongly supportive of Dbp5 functioning in tRNA export within the nucleus.
- This defect was largely rescued in the *dbp5-L12A/R423A* double mutant (43 ± 7), returning to levels that were not significantly different from the d*bp5-L12A* mutant alone (Figure 5A).
- In any of these cases, introduction of the L12A mutation would rescue tRNA export defects, as was observed. This would occur through increased nuclear levels of Dbp5 above the threshold needed to support tRNA export, including a point where the frequency of binding events ensures the likelihood of binding being coupled to an ATPase hydrolysis event.
- Figure 5. Dbp5 supports re-export of mature tRNAs following nutritional stress. A. Localization of tRNA^Tyr^ determined by FISH at 25°C in integrated and untagged *DBP5, dbp5-L12A, dbp5-R423A,* and *dbp5-L12A/R423A* strains prior to (pre), during a 10 min starvation for amino acids (-AA), and 15 min after reintroduction of amino acids through addition of rich media (re-feed).

Corrected:

- A strain expressing GFP-Dbp5^L12A/R423A^ is viable and there is an increased nuclear pool of Dbp5 as compared to R423A (Figure 4—﻿figure supplement 1A); however, the untagged and integrated version of *dbp5-L12A/R423A* exhibited mRNP export defects at both 25°C and 37°C, and a strong temperature-dependent growth defect at 37°C (Figure 4—﻿figure supplement 1B-C). This result indicates that further limiting the pool of cytoplasmic Dbp5 in the context of the R423A mutation alters mRNP export. Using the double mutant, northern blotting was performed with probes against the tRNA^Ile^_UAU_ at 37°C (Figure 4D). As compared to *dbp5-R423A* (1.5 ± 0.2), accumulation of the intron containing tRNA intermediate was similar in the untagged and integrated *dbp5-L12A/R423A* (1.9 ± 0.5), suggesting that depleting the cytoplasmic pool of Dbp5 through inclusion of the L12A mutation did not further impact tRNA processing, in contrast to what is seen with mRNP export. Due to the presence of mRNP export defects at 25°C, this mutant was not used to further interrogate the role of Dbp5 in tRNA export. Combined, the SGA, northern, and in situ data employing the *dbp5-L12A* and *dbp5-R423A* mutations to alter Dbp5 nuclear levels are strongly supportive of Dbp5 functioning in tRNA export within the nucleus, while facilitating mRNP export in the cytoplasmic compartment (e.g. at the cytoplasmic face of NPCs).
- This sentence was removed.
- This sentence was removed.
- Figure 5. Dbp5 supports re-export of mature tRNAs following nutritional stress. A. Localization of tRNA^Tyr^ determined by FISH at 25°C in integrated and untagged *DBP5, dbp5-L12A*, **and** *dbp5-R423A* strains prior to (pre), during a 10 min starvation for amino acids (-AA), and 15 min after reintroduction of amino acids through addition of rich media (re-feed).

2. Figure 4 panel D and Figure 4—figure supplement 1 panels B and C have been updated with data showing the correct phenotype for *dbp5-L12A-R423A*. Data from *dbp5-L12A-R423A* has been removed from Figure 5 panel A. We have elected to remove and not replace this data as the tRNA processing defects and poly(A)-RNA accumulation phenotypes of *dbp5-L12A-R423A* no longer make this a logical experiment. See below for original and corrected figures.

The corrected Figure 4 is shown here:

**Figure 1:**
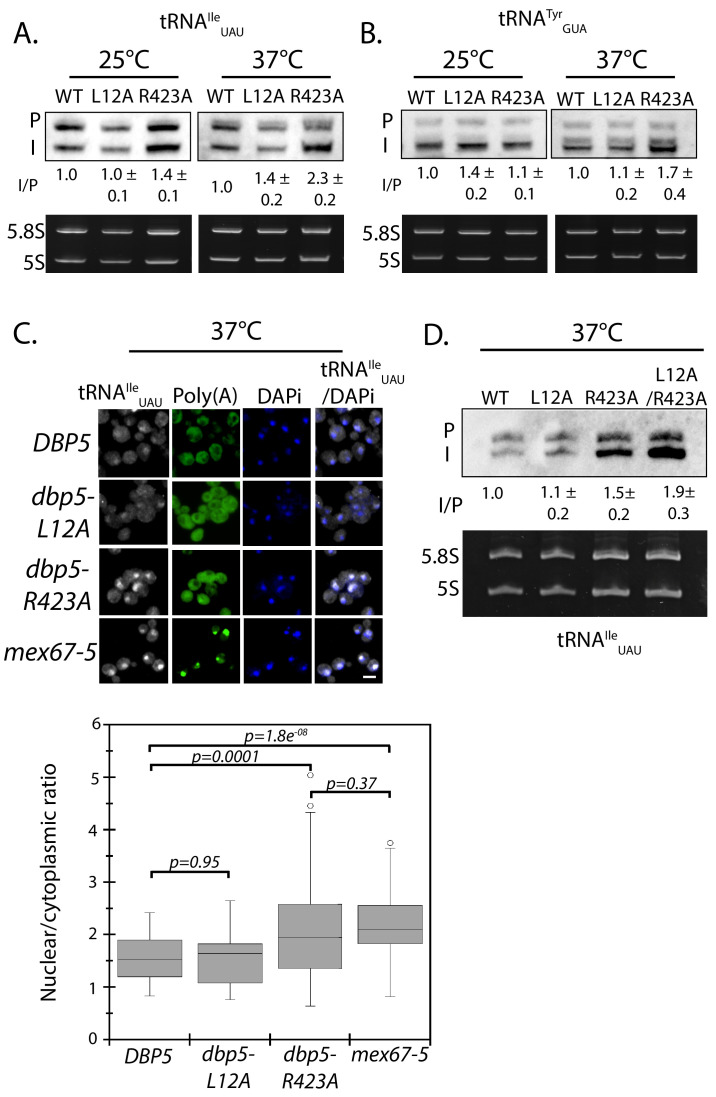


The originally published Figure 4 is also shown for reference:

**Figure 2:**
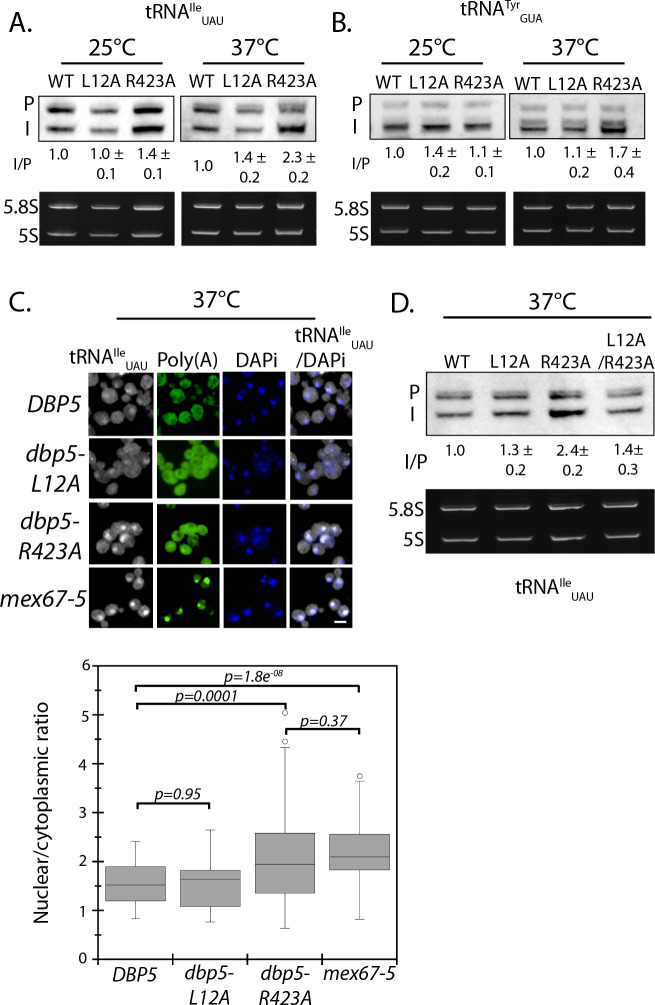


The corrected Figure 4—figure supplement 1 is shown here:

**Figure 3:**
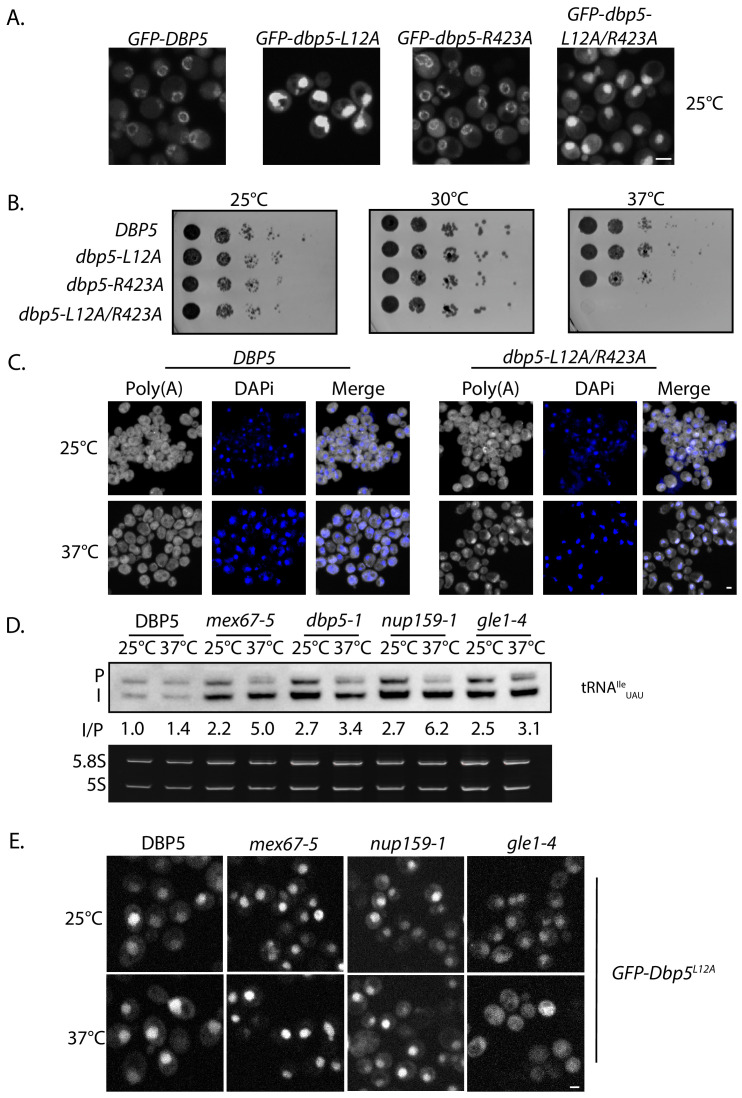


The originally published Figure 4—figure supplement 1 is also shown for reference:

**Figure 4:**
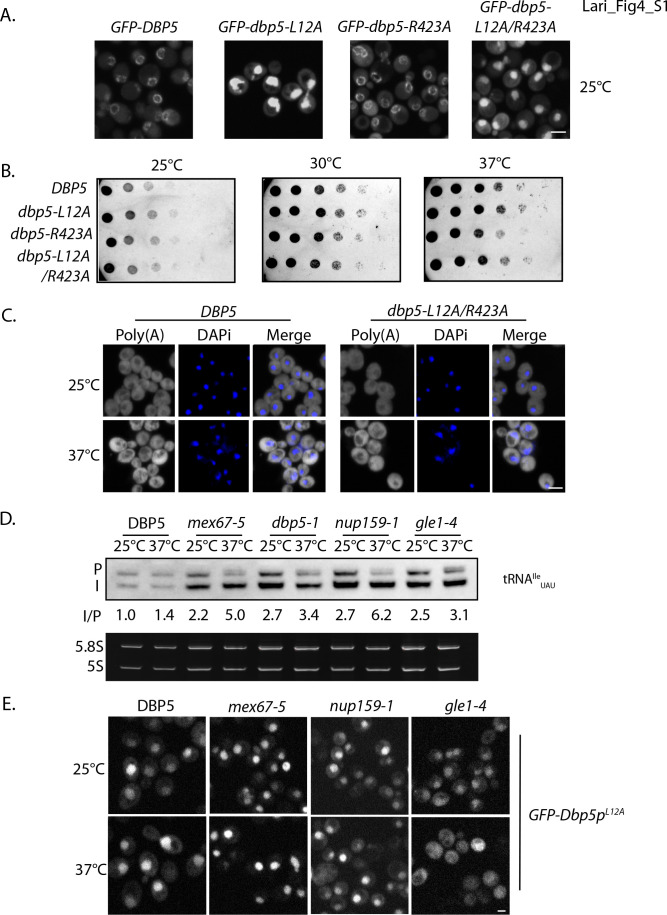


The corrected Figure 5 is shown here:

**Figure 5:**
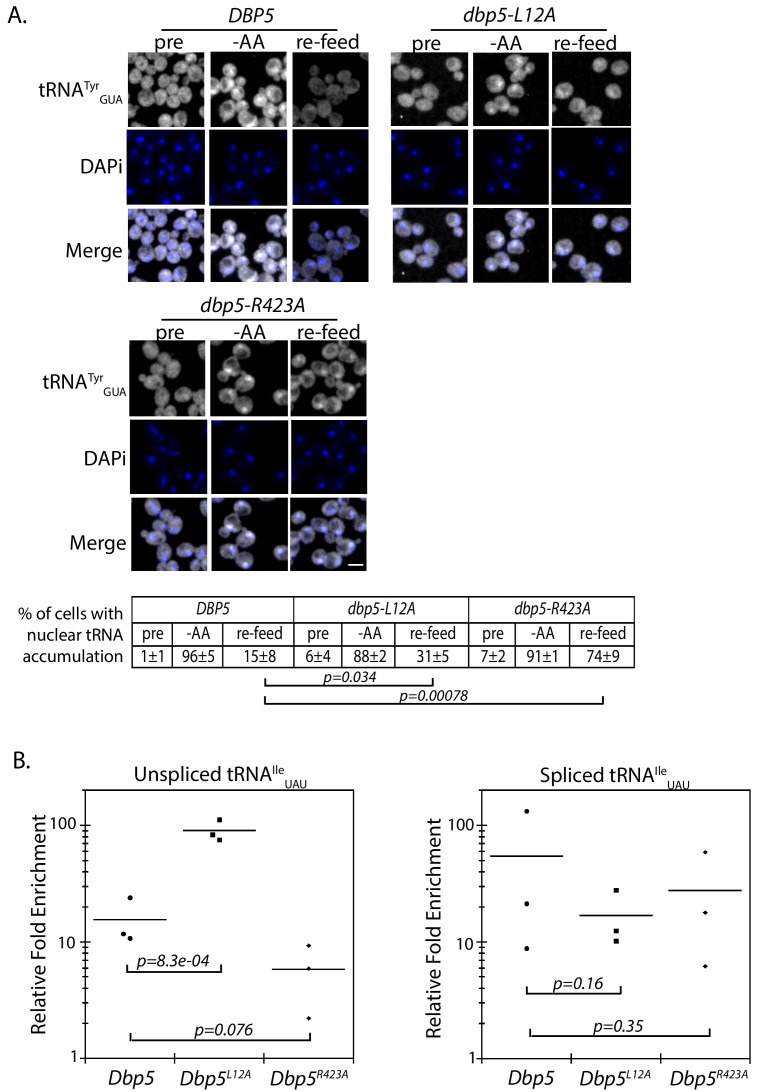


The originally published Figure 5 is also shown for reference:

**Figure 6:**
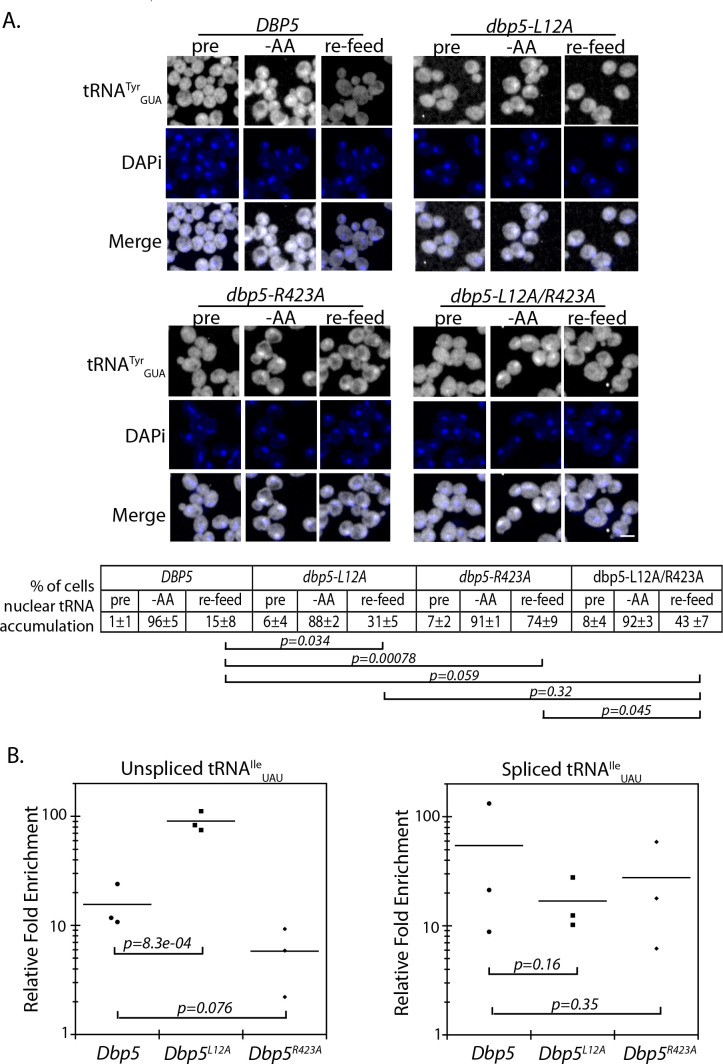


The article has been corrected accordingly.

